# Novel expression pattern of neuropeptide Y immunoreactivity in the peripheral nervous system in a rat model of neuropathic pain

**DOI:** 10.1186/s12990-015-0029-y

**Published:** 2015-05-27

**Authors:** Claire Magnussen, Shih-Ping Hung, Alfredo Ribeiro-da-Silva

**Affiliations:** Department of Pharmacology and Therapeutics, McGill University, McIntyre Medical Building, 3655 Promenade Sir William Osler, Room 1215, Montreal, Quebec H3G 1Y6 Canada; Alan Edwards Centre for Research on Pain, McGill University, Montreal, Quebec H3A 0G1 Canada; Department of Anatomy and Cell Biology, McGill University, Montreal, Quebec H3A 0C7 Canada

**Keywords:** Neuropeptide tyrosine, NPY, Innervation, NF200, CGRP, IB4, Sympathetic nervous system, Skin, Mental nerve, Trigeminal, Pain

## Abstract

**Background:**

Neuropeptide Y (NPY) has been implicated in the modulation of pain. Under normal conditions, NPY is found in interneurons in the dorsal horn of the spinal cord and in sympathetic postganglionic neurons but is absent from the cell bodies of sensory neurons. Following peripheral nerve injury NPY is dramatically upregulated in the sensory ganglia. How NPY expression is altered in the peripheral nervous system, distal to a site of nerve lesion, remains unknown. To address this question, NPY expression was investigated using immunohistochemistry at the level of the trigeminal ganglion, the mental nerve and in the skin of the lower lip in relation to markers of sensory and sympathetic fibers in a rat model of trigeminal neuropathic pain.

**Results:**

At 2 and 6 weeks after chronic constriction injury (CCI) of the mental nerve, *de novo* expression of NPY was seen in the trigeminal ganglia, in axons in the mental nerve, and in fibers in the upper dermis of the skin. In lesioned animals, NPY immunoreactivity was expressed primarily by large diameter mental nerve sensory neurons retrogradely labelled with Fluorogold. Many axons transported this *de novo* NPY to the periphery as NPY-immunoreactive (IR) fibers were seen in the mental nerve both proximal and distal to the CCI. Some of these NPY-IR axons co-expressed Neurofilament 200 (NF200), a marker for myelinated sensory fibers, and occasionally colocalization was seen in their terminals in the skin. Peptidergic and non-peptidergic C fibers expressing calcitonin gene-related peptide (CGRP) or binding isolectin B4 (IB4), respectively, never expressed NPY. CCI caused a significant *de novo* sprouting of sympathetic fibers into the upper dermis of the skin, and most, but not all of these fibers, expressed NPY.

**Conclusions:**

This is the first study to provide a comprehensive description of changes in NPY expression in the periphery after nerve injury. Novel expression of NPY in the skin comes mostly from sprouted sympathetic fibers. This information is fundamental in order to understand where endogenous NPY is expressed, and how it might be acting to modulate pain in the periphery.

## Introduction

In animal models of neuropathic pain, the landscapes of the central (CNS) and peripheral nervous systems (PNS) are vastly modified with some of the most striking changes occurring in the skin, where a noxious stimulus is first detected. Injury to the trigeminal or sciatic nerve causes a loss of sensory fibers in the skin, including neurofilament 200 (NF200)-immunoreactive (IR) myelinated and calcitonin gene-relate peptide (CGRP)-IR fibers, that transmit touch and nociceptive information, respectively [1–5]. In these models, sympathetic fibers invade the upper dermis of the skin, a region from where they are normally devoid, and form close associations with sensory fibers [2, 3, 6, 7]. These gross anatomical changes are often accompanied by alterations in neurochemistry, including changes in the peptide content of primary afferents [8, 9].

Neuropeptide tyrosine, also named neuropeptide Y (NPY), is a tyrosine-rich peptide that is implicated in many homeostatic roles including the modulation of pain. In mammals, it acts by binding to its receptors, a family of five G-protein coupled receptors (Y1, Y2, Y4, Y5 and Y6), with the Y1 and Y2 receptors being most heavily connected to pain mechanisms [10]. Pharmacologically, NPY delivered intrathecally reduces tactile and thermal allodynia that develop following the spared nerve injury (SNI) model of neuropathic pain [11], and conditional knockdown of NPY either prior to, or after SNI, significantly increases both mechanical and cold allodynia [12]. To complicate matters, NPY administered subcutaneously into the paw after partial sciatic nerve ligation (PSNL) exacerbates mechanical and thermal allodynia [13]. While it is clear that NPY modulates pain, its effect might be determined by its site of action.

Due to its diverse physiological roles, NPY is distributed throughout the CNS and PNS [14]. It is colocalized with norepinephrine in postganglionic sympathetic neurons [15, 16] and is especially abundant in the superficial dorsal horn (laminae I and II) [17, 18]. Following nerve injury, large dorsal root ganglia (DRG) cell bodies, which normally do not express NPY, upregulate NPY [19–26]. In contrast, C fibers almost never express NPY after injury [27]. Parallel observations were seen after transection of the mental nerve, a branch of the trigeminal nerve that innervates the lower lip. [26].

NPY also accumulates in the neuroma that develops proximal to sciatic nerve transection [28–30], but whether NPY is transported through the site of injury remains unknown. Furthermore, the innervation pattern of NPY in the skin has never been described in a model of chronic pain. To this end, NPY expression was examined in the PNS in a rat model of trigeminal neuropathic pain in which a modified chronic constriction injury (CCI) is applied to the mental nerve [3]. NPY immunoreactivity was studied in the trigeminal ganglia, the mental nerve and in the skin of the lower lip, in relation to markers of sensory and sympathetic fibers, 2 and 6 weeks after CCI. We show that newly synthesized NPY is transported in sensory neurons through the site of injury to the skin where it can be found in both sprouted sympathetic fibers and occasionally in NF200 fibers.

## Results

### Bilateral chronic constriction injury of the mental nerve induced mechanical allodynia in the lower lip

**Fig. 1:**
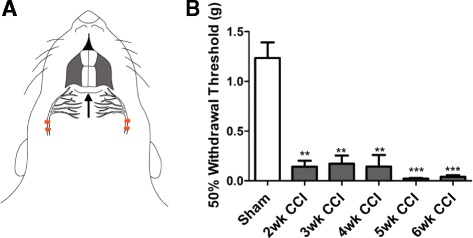
Bilateral chronic constriction injury of the mental nerve induced mechanical allodynia in the lower lip. **a**: Trigeminal neuropathic pain was induced in the lower lip of the rat by applying two loose ligatures, shown in red, around each mental nerve. To assess the presence of mechanical allodynia, von Frey fibers were applied perpendicularly to the lower lip along the midline in unrestrained animals. The black arrow shows the region where the von Frey fibers were applied. **b**: Sham surgery resulted in no change in mechanical thresholds at any time point, so were pooled together. When compared to shams, CCI produced a significant reduction in 50 % withdrawal thresholds to von Frey hairs 2–6 weeks after surgery (n = 8). Each point represents the mean ± SEM **p < 0.01, ***p < 0.001 by 1way ANOVA with Dunnett’s post hoc test

Trigeminal neuropathic pain was induced by applying a modified version of the chronic constriction injury (CCI) to both mental nerves which innervate the rat lower lip (Fig. 1a). Sham surgery resulted in no change in mechanical thresholds at any time point, so were pooled together. CCI animals developed mechanical allodynia, seen as significant reduction in 50 % withdrawal threshold compared to sham animals, starting 2 weeks and persisting to 6 weeks after surgery (Fig. 1b).

### *De novo* expression of NPY in the trigeminal ganglia, the skin and the mental nerve 2 and 6 weeks after CCI

**Fig. 2:**
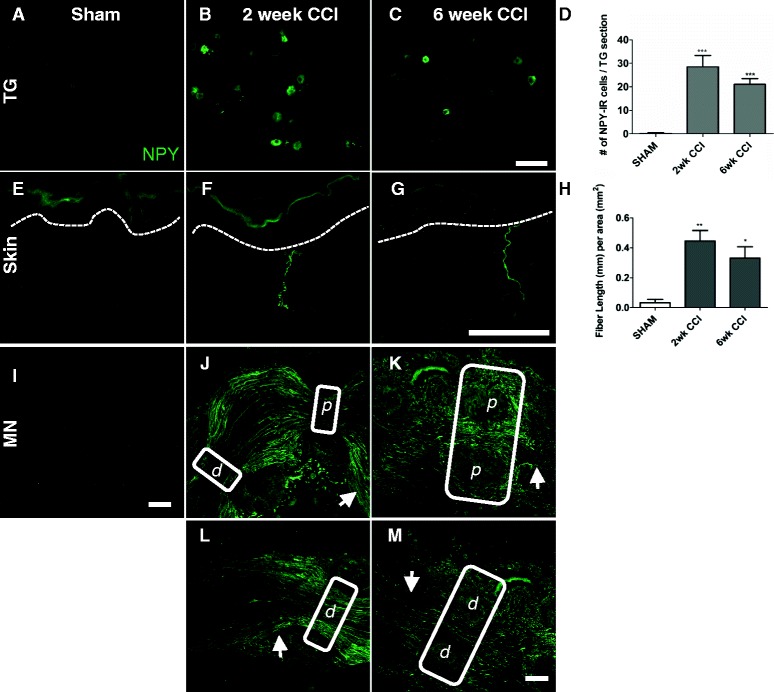
*De novo* expression of NPY in the trigeminal ganglia, skin and mental nerve 2 and 6 weeks after CCI. NPY-IR cell bodies are absent from the trigeminal ganglia (TG) of sham animals (**a**) but are abundant 2 (**b**) and 6 weeks (**c**) after CCI. Quantification of the number of NPY-IR cell bodies per TG section is shown in **d** (n = 6). NPY-IR fibers are absent from the upper dermis of sham lip skin (**e**), but are present in this region 2 (**f**) and 6 weeks (**g**) after CCI. Dashed lines represent the border between the epidermis and upper dermis. Quantification of NPY fiber length (mm) per area of upper dermis (mm^2^) is shown in **h** (n = 5–6). Parasagittal sections of mental nerve (MN) from sham (**i**) and CCI animals 2 (**j**, **l**) and 6 weeks (**k**, **m**) after surgery. NPY fibers are absent from the MN of sham animals (**i**). In CCI animals, proximal and distal ligations are identified by rectangles labelled with ***p*** and ***d***, respectively and arrows identify the regions of the nerve proximal (**j**, **k**) and distal (**l**, **m**) to the ligations. Two weeks after CCI, NPY-IR fibers are present in the nerve proximal to the ligations (**j**), between the ligations (**j**) and distal to the ligations (**l**). Six weeks after CCI, NPY-IR fibers are present in the nerve proximal to the ligations (**k**), between the ligations (**k**) and distal to the ligations (**m**). Six week sham animals were used to show representative images and data from 2 and 6 week sham animals were pooled for quantification. Values represent mean ± SEM *p < 0.05, ** < 0.01, ***p < 0.001 by 1 way ANOVA with Dunnett’s post hoc test. Scale bars = 100 μm. *TG*: trigeminal ganglia, *MN*: mental nerve, *p*: proximal ligature, *d*: distal ligature

CCI, in addition to causing mechanical allodynia, produced a striking *de novo* upregulation of NPY in the trigeminal ganglia, the mental nerve and in the skin of the lower lip, 2 and 6 weeks after injury, as observed using immunohistochemistry (Fig. 2). In sham animals, sensory cell bodies in the trigeminal ganglia almost never expressed NPY (Fig. 2a), however after CCI, NPY immunoreactivity was observed in many neurons (Fig. 2b, c). Whereas shams had an average of 0.2 ± 0.2 NPY-IR cells per section of trigeminal ganglia, CCI animals had significantly more NPY-IR cell bodies at 2 (28.6 ± 4.8) and 6 weeks (21 ± 2.5) after surgery (Fig. 2d). NPY immunoreactivity was observed in cells with a mean diameter of 36.4 ± 0.6 μm (2 week CCI) and 34.7 ± 1.4 μm (6 week CCI), indicating that it predominantly became expressed in large trigeminal ganglion neurons.

In the skin of the lower lip, NPY immunoreactivity was also increased (Fig. 2e–g). We focused our investigation on the upper dermis of the skin as innervation changes in this area have been widely reported in many models of chronic pain [1–3, 6, 7, 31, 32]. In shams, almost no NPY immunoreactivity was observed (Fig. 2e), however after CCI, NPY-IR fibers were readily seen in the upper dermis (Fig. 2f, g). NPY fiber density, as measured by fiber length (mm) per area of upper dermis (mm^2^), was significantly greater than in sham at both time points after CCI (Fig. 2h; Sham 0.032 ± 0.02; 2 week CCI 0.4 ± 0.07, p < 0.01; 6 week CCI 0.3 ± 0.07, p < 0.05).

NPY immunoreactivity was not seen in the mental nerve of sham animals (Fig. 2i), but 2 and 6 weeks after CCI, many NPY-IR fibers were found proximal to the first ligature. These fibers passed through both the first and second ligatures (Fig. 2j, k), and could be seen running in the nerve distal to injury (Fig. 2l, m).

### NPY was expressed in Fluorogold labelled mental nerve cell bodies after CCI

**Fig. 3:**
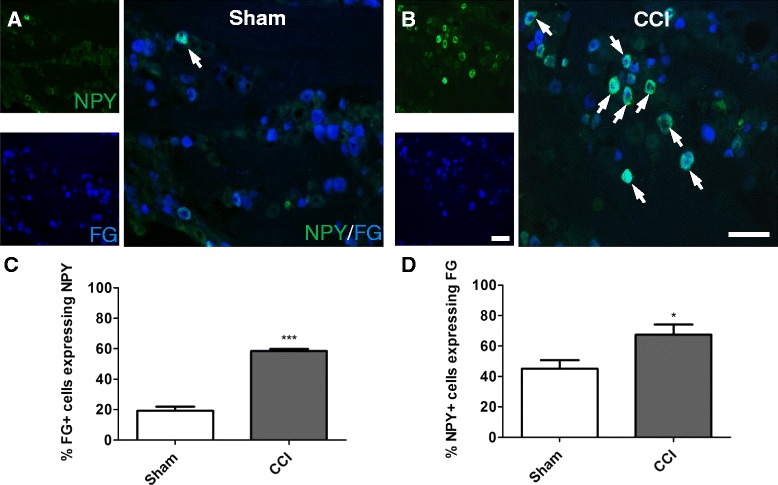
NPY was expressed in Fluorogold labelled mental nerve cell bodies after CCI. Fluorogold (FG), in blue, was used to retrogradely label the cell bodies of mental nerve sensory neurons. NPY immunoreactivity is shown in green. Signals are shown separately in small panels, and together in the adjacent larger panel. Colocalization of the two signals is shown in turquoise. Few NPY cells were seen in sham animals (**a**), but in CCI animals many NPY cells colocalized with FG (**b**). Arrows indicate examples of colocalization. The quantification of the percentage of FG cells that expressed NPY is shown in **c**. The quantification of the percentage of NPY cells that expressed FG is shown in **d**. Values represent mean ± SEM (n = 6); *p < 0.05, ***p < 0.001 by Student’s *t*-test. Scale bar = 100 μm

The increase in NPY immunoreactivity in the trigeminal ganglia after CCI was evident, but was this upregulation occurring specifically in the cell bodies of mental nerve sensory neurons, or was it occurring through the entire trigeminal ganglia? To address this question, Fluorogold was injected into the mental nerve following sham or CCI surgery to retrogradely label the cell bodies of the mental nerve sensory neurons. NPY expression in these cells was evaluated 1 week after injection. Unlike a normal sham surgery which does not provoke the upregulation of NPY, injection of Fluorogold was sufficient to produce a small increase in NPY immunoreactivity in the ganglia of sham animals (Fig. 3a). However, after CCI, a significantly higher percentage of Fluorogold cells expressed NPY (Fig. 3b, c; Sham 19.2 ± 2.7 %; CCI 58.5 ± 1.3 %, p < 0.001). In both sham and CCI, NPY was not expressed exclusively by Fluorogold-labelled cells (Fig. 3d; Sham 45.0 ± 5.7 %; CCI 67.4 ± 5.7 %, *p* < 0.05).

### NPY was expressed by some NF200-IR sensory neurons after CCI

**Fig. 4:**
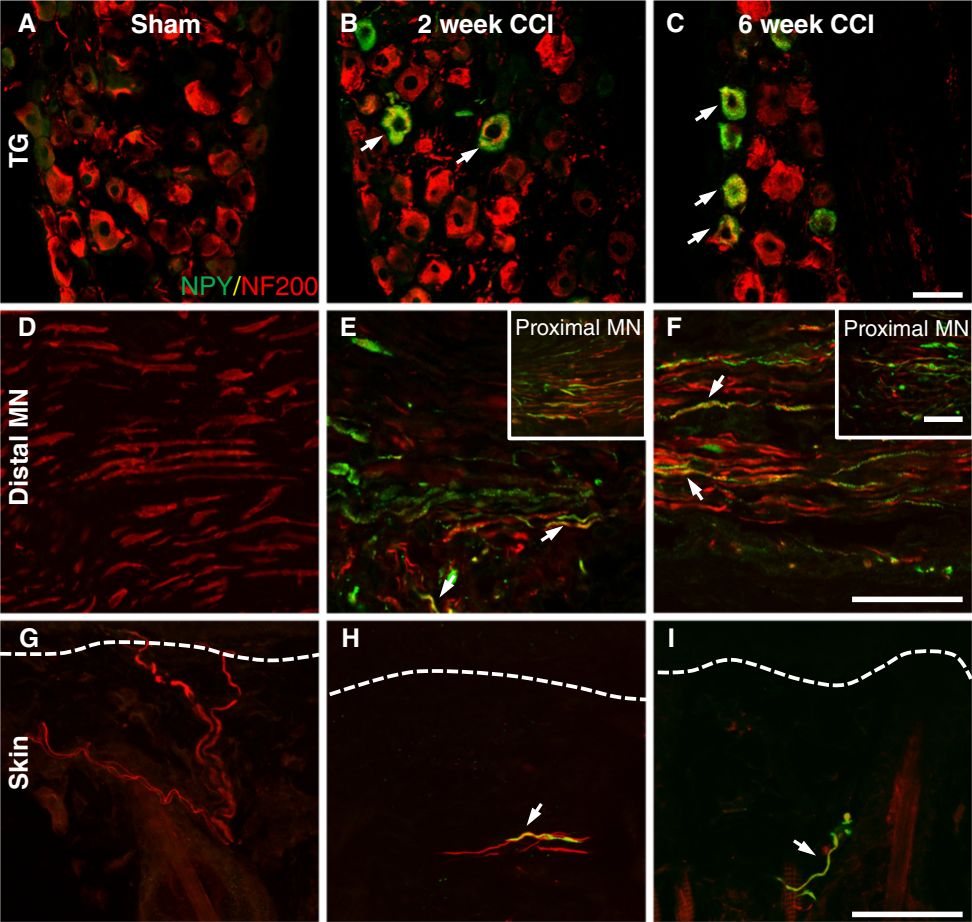
NPY was expressed by some NF200-IR sensory neurons after CCI. The presence of NPY immunoreactivity (in green) in NF200-positive sensory fibers (in red) was assessed in sham animals and in CCI animals 2 and 6 weeks after injury in the trigeminal ganglia (TG), the mental nerve (MN) and in the upper dermis of the skin. Colocalization between these two markers is shown in yellow and highlighted with an arrow. NPY was not seen in the TG of sham animals (**a**), but was expressed in some NF200-IR cell bodies 2 (**b**) and 6 weeks (**c**) after CCI. The percentage of NF200-IR cells that expressed NPY was quantified (Sham 0 ± 0; 2 week CCI 28.7 ± 5.4 %; 6 week CCI 21.1 ± 2.5 %; n = 6). The percentage of NPY-IR cells that expressed NF200 was quantified (Sham 0 ± 0; 2 week CCI 57.5 ± 3.8 %; 6 week CCI 48.5 ± 5.1 %; n = 6). No NPY-IR fibers were seen in the MN of sham animals (**d)**. Two (**e**) and 6 weeks (**f**) after CCI some NF200 fibers distal to the ligation expressed NPY. NPY was also seen proximal to the ligation (inset in **e-f**). In the skin, NF200 fibers were present in the upper dermis of sham animals (**g**). NF200 fiber density was reduced in the skin after CCI and some NF200 fibers expressed NPY immunoreactivity 2 (**h**) and 6 weeks (**i**) after CCI. Dashed lines represent the border between the epidermis and the upper dermis. In all cases, 6 week sham animals were used as representative images and data from 2 and 6 week sham animals were pooled for quantification. Scale bar = 50 μm *TG*: trigeminal ganglia, *MN*: mental nerve

**Fig. 5:**
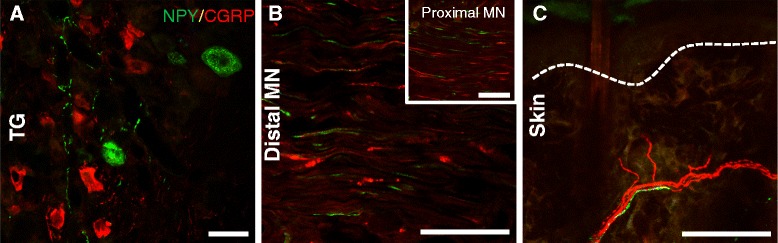
NPY was not expressed by CGRP-IR sensory neurons after CCI. The presence of NPY (in green) in CGRP-positive primary afferents (in red) was assessed after CCI in the trigeminal ganglia (TG), the mental nerve (MN) and in the upper dermis of the skin. Representative images were taken from a CCI animal 6 weeks after surgery, but the same pattern was observed at 2 weeks (data not shown). NPY immunoreactivity was expressed by some cell bodies in the TG as well as by fibers, but these never co-expressed CGRP (**a**). NPY-IR fibers were seen in the mental nerve both proximal (inset) and distal to the CCI, but these fibers never co-expressed CGRP (**b**). In the skin, NPY-IR fibers never colocalized with CGRP, but were sometimes seen in close proximity (**c**). Dashed lines represent the border between the epidermis and the upper dermis. Scale bar = 50 μm *TG*: trigeminal ganglia, *MN*: mental nerve

**Fig. 6:**
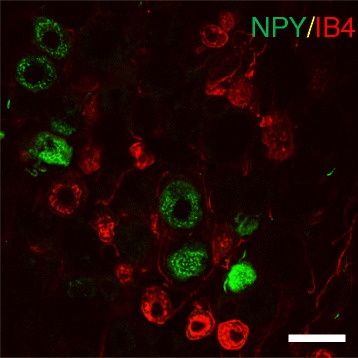
NPY was not expressed by IB4 binding sensory neurons after CCI. The presence of NPY (in green) in IB4 binding primary afferents (in red) was assessed in the trigeminal ganglia (TG) after CCI. This representative image was taken from a CCI animal 6 weeks after surgery, but the same pattern was observed at 2 weeks (data not shown). NPY immunoreactivity was expressed by some cell bodies in the TG, but these did not bind IB4. Scale bar = 50 μm

To determine in which population of sensory fibers NPY became expressed in after nerve injury, NPY immunoreactivity was studied in relation to NF200 (Fig. 4), a marker of myelinated fibers, CGRP (Fig. 5), a marker for small diameter peptide containing nociceptive fibers and IB4 (Fig. 6), a marker for small diameter non-peptidergic nociceptive fibers. Following CCI, there was a *de novo* expression of NPY in NF200-IR trigeminal ganglia neurons (Fig. 4a–c). In sham animals, no NPY-IR cell bodies were seen (Fig. 4a), however 2 and 6 weeks after CCI, 28.7 ± 5.4 % and 21.1 ± 2.5 % of NF200-IR cells expressed NPY, respectively (n = 6). Not all NPY-IR cell bodies expressed NF200 after CCI, with about 50 % of NPY-IR cells also NF200-IR (2 week CCI 57.5 ± 3.8 %; 6 week CCI 48.5 ± 5.1 %). This NPY seemed to be transported peripherally in the axons of these neurons, as some NF200 fibers in the mental nerve expressed NPY both proximal (Fig. 4ef–f inset) and distal to the ligation (Fig. 4e, f). In the skin, NF200 fiber density in the upper dermis was significantly reduced after CCI compared to sham (Fig. 4g), however occasionally NF200 fibers expressed NPY in the skin of CCI animals (Fig. 4h-i). After extensive qualitative examination, the amount of colocalization between NF200 and NPY appeared to be greatest in the trigeminal ganglia, with less colocalization observed in the mental nerve and even lesser colocalization in the skin.

### NPY was not expressed by small CGRP-IR or IB4-binding sensory neurons after CCI

In contrast to NF200, CGRP-IR neurons never expressed NPY after CCI (Fig. 5). Images from a 6 week CCI were chosen for illustration, and it is evident that CGRP and NPY were never found together in the trigeminal ganglia (Fig. 5a) nor in the mental nerve proximal (inset) or distal to the ligation (Fig. 5b). The same was true 2 weeks after CCI (data not shown). In the skin, NPY and CGRP fibers never colocalized, however they could be seen to run alongside one another (Fig. 5c). Because NPY is almost never upregulated in C fibers after injury [27], IB4-binding non-peptidergic neurons were not expected to express NPY. As predicted, IB4+ cell bodies in the trigeminal ganglia did not express NPY after CCI (Fig. 6).

### CCI induced a sprouting of sympathetic fibers into the upper dermis of the skin, and these fibers often expressed NPY

**Fig. 7:**
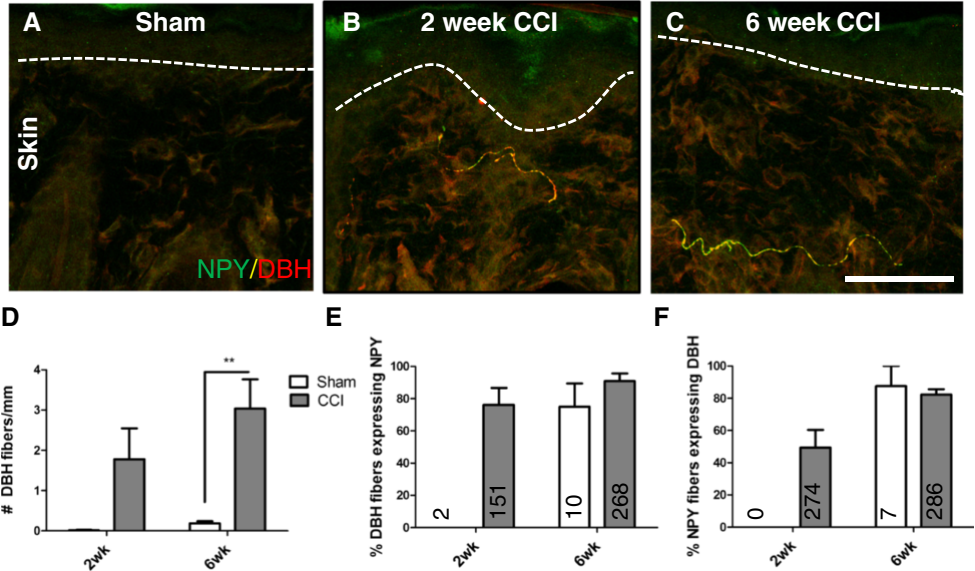
CCI induced a sprouting of sympathetic fibers into the upper dermis of the skin, and these fibers often expressed NPY. Sympathetic fibers labelled with DBH (in red), were rarely observed in the upper dermis of the skin in 6 week sham animals (**a**). 2 (**b**) and 6 (**c**) weeks after CCI, sympathetic fibers sprouted into the upper dermis, and many expressed NPY. The quantification of the number of DBH fibers per mm of upper dermis is shown in (**d**). The quantification of the percentage of DBH fibers that expressed NPY is shown in **e** and the values written on the graphs indicate the absolute number of sprouted sympathetic fibers counted across animals. The quantification of the percentage of NPY fibers that expressed DBH is shown in **f** and the values written on the graphs indicate the absolute number of NPY-positive fibers counted across animals. Dashed lines represent the border between the epidermis and the upper dermis. **p < 0.01 by 2 way ANOVA with Bonferroni post hoc (n = 6). Scale bar = 50 μm

Sympathetic fibers normally express DBH, as well as NPY. In the trigeminal ganglia of both sham and CCI animals, sympathetic fibers were occasionally seen either around blood vessels or along the capsule of the ganglia, and these fibers colocalized DBH and NPY (data not shown). In the skin, sympathetic fibers were almost never seen in the upper dermis of sham animals (Fig.7a), but 2 and 6 weeks after CCI, they sprouted into this territory and many expressed NPY (Fig. 7b, c). While there was a large trend towards an increase in sympathetic fibers 2 weeks after CCI, this was significant at 6 weeks when the number of DBH-IR fibers increased from 0.18 ± 0.05/mm in sham to 3.0 ± 0.72/mm in CCI, p < 0.01 (Fig. 7d). Many, but not all, sprouted DBH-IR fibers expressed NPY, and the percentage of colocalization between these two markers increased over time in CCI animals (Fig. 7e; 2 week Sham 0 ± 0 %, 2 week CCI 76.1 ± 10.6 %; 6 week Sham 75 ± 14.4 %, 6 week CCI 90.8 ± 4.8 %). While it was possible to calculate a percentage of colocalization between these two markers in all 4 groups of animals, the absolute number of sprouted sympathetic fibers varied considerably between groups with 2, 151, 10 and 256 fibers in the upper dermis of 2 week sham, 2 week CCI, 6 week sham and 6 week CCI animals, respectively. After CCI, not all NPY-IR structures were sympathetic fibers, and this is reflected by the percentage of NPY fibers that expressed DBH (Fig. 7f; 2 week Sham 0 ± 0 %, 2 week CCI 49.4 ± 11.0 %; 6 week Sham 87.5 ± 12.5 %, 6 week CCI 82.4 ± 3.2 %). The absolute number of NPY fibers in the upper dermis per group was 0, 274, 7 and 286 in 2 week sham, 2 week CCI, 6 week sham and 6 week CCI, respectively.

## Discussion

This study is the first to demonstrate significant increases in NPY immunoreactivity distal to a site of peripheral nerve injury. Our results suggest that newly synthesized NPY in the trigeminal ganglia is transported in the axons of many sensory fibers, including a few remaining NF200 primary afferents, through the lesion in the mental nerve, to their terminations in the skin. However, most of the *de novo* NPY in the upper dermis of the skin originated from sprouted sympathetic fibers, which do not travel in the mental nerve [6].

The *de novo* expression of NPY in large diameter cell bodies is consistent across many different models of neuropathic [19, 22, 23, 25, 33] and trigeminal neuropathic pain [26, 34–36]. Here we report that NPY became expressed in trigeminal ganglia neurons with an average diameter of 36.4 μm at 2 weeks after CCI of the mental nerve, consistent with what has been reported after mental nerve transection [26]. Based on the size of these NPY-IR cell bodies, it is not surprising that roughly 50 % also expressed NF200, a reliable marker of Aβ and Aδ fibers [27]. Based on existing literature, C fibers, with small diameter cell bodies, were not expected to express NPY after CCI [27], and as anticipated, NPY never colocalized with CGRP or IB4 in the ganglia.

The exact triggers that result in the *do novo* expression of NPY by sensory neurons remain unknown. However, it has been proposed that nerve trauma, and not pain *per se*, might be the precipitating factor since painful inflammation does not increase DRG NPY immunoreactivity [23, 37]. In models of neuropathic pain, the expression of NPY seems to correlate with the extent of nerve injury [36, 38, 39] and in our hands, a small trauma, like the one that occurred when Fluorogold was injected into the nerve, triggered a limited NPY expression in the ganglia of sham animals. 1 week after CCI, the majority (67 %) of NPY-IR cells also expressed Fluorogold, indicating a certain spatial specificity to this response. Because Fluorogold is not taken up by intact axons at nonterminal sites [40], this technique was used to retrogradely label injured neurons, and underestimates the total number of mental nerve sensory neurons. While we and the literature agree that NPY is expressed by injured sensory neurons [21, 25, 41], our results suggest that a smaller proportion of uninjured neurons also begin to express NPY after CCI, in agreement with Ma and Bisby [41]. This is not the case after median nerve transection where only 1 % of uninjured neurons expressed NPY [25].

We suggest that these uninjured neurons are the first to transport NPY through the site of injury in the mental nerve. While many NPY-IR fibers are seen in the nerve, only some co-express NF200. In fact, the colocalization between NF200 and NPY appears to be the greatest in the ganglia, with lower levels observed in the nerve and in the skin, respectively. This is unlikely due to slow shipment of the peptide to the periphery as NPY, synthesized in the cell body, is sent by fast axonal transport at a rate of approximately 9 mm/h [42]. Given that its mRNA and protein are increased as early as 3 days after nerve injury [25, 35, 43], NPY could theoretically reach the terminals of NF200 fibers only 4 days after CCI. Instead the reduced colocalization between these two markers in distal nerves is most likely explained by the findings that 90 % of A fibers did not conduct impulses through the site of injury 3 days after CCI [44] and that NF200 fiber density in the skin is drastically reduced up to 16 weeks after CCI [1]. As NPY immunoreactivity remains increased in the ganglia up to 24 weeks after nerve injury [21], it is possible that greater colocalization between NF200 and NPY could be observed distal to CCI at these later time points when there is more NF200 fiber recovery. The induction of NPY in these sensory fibers may reflect a mechanism for altered neuronal activity after injury. Indeed, following spinal nerve ligation, Aβ DRGs from NPY overexpressing mice displayed longer refractory periods and had higher voltage thresholds compared to wild type animals, indicating that NPY in surplus can decrease the responses of these fibers to nerve injury [45].

While NF200 fibers represent a minor source of *de novo* NPY in the skin, sprouted sympathetic fibers, which invade the upper dermis at 2 weeks [3, 7] represent the major source. The colocalization between DBH and NPY increased over time, and by 6 weeks after CCI approximately 90 % of DBH-IR sprouted sympathetic fibers expressed NPY. NPY is co-expressed with norepinephrine in many sympathetic fibers [42, 46, 47], however there exists heterogeneity within this population and not all fibers contain NPY [16]. In our model, previous studies have shown evidence that these ectopic fibers migrate from the lower dermis [3, 6], where sympathetic fibers predominantly innervate vasculature and almost always contain NPY (unpublished observations). We are therefore not surprised that these sprouted sympathetic fibers mostly maintain their original phenotype. Normally, NPY is preferentially released following high frequency stimulation and leads to long lasting vasoconstriction [48], however because these sprouted sympathetic fibers are most often not associated with blood vessels, they may function in a different capacity after nerve injury. Finally, because our data indicate that total NPY immunoreactivity in the upper dermis cannot be explained alone by its expression in sympathetic fibers, NPY should not be used as a reliable marker of this fiber population after nerve injury.

In this study, we show two novel sources of NPY in the upper dermis of the skin after CCI, however how this increased NPY contributes to pain is difficult to directly examine. Mental nerve CCI resulted in mechanical allodynia that lasted from 2 to 6 weeks, the last time point tested. Because neither the withdrawal thresholds nor levels of NPY changed significantly during this time, we are unable to correlate alterations in NPY immunoreactivity in the periphery with pain behaviour. In the literature however there is considerable evidence in support of the idea that upregulation of NPY by sensory neurons represents an adaptive mechanism that counteracts the evolution of neuropathic pain [27]—for reviews see [10, 49]. Indeed, conditional knockdown of NPY resulted in a significant increase in the intensity and duration of mechanical and thermal hypersensitivity after SNI suggesting that NPY inhibits pain [12].

In the periphery, the situation might be different, with NPY being pronociceptive through its action at Y2 receptors, or having mixed effects through the Y1 receptor. Subcutaneous injections of NPY and an NPY Y2 receptor agonist into the paw after PSNL exacerbated mechanical allodynia and thermal hyperalgesia [13]. Could NPY released from these sprouted sympathetic fibers worsen pain? A novel physical proximity exists between sprouted sympathetic fibers and sensory fibers after nerve injury [2, 3, 6, 7], and we have shown here that NPY fibers run in bundles with CGRP fibers in the skin. It is conceivable that NPY released from these fibers could directly modulate the activity of primary afferents, known to express Y1 and Y2 [50–53]. Indeed, NPY through its actions on Y2 increased the excitability of sensory neurons [54]. Alternatively, NPY might act directly on these sprouted sympathetic fibers, through Y2 autoreceptors [55], to modulate the release of excitatory norepinephrine, or even NPY itself [13, 56, 57]. NPY in the skin can also act on Y1 receptors, and it has been shown that an intraplantar injection of a Y1 agonist worsens mechanical hyperalgesia, while concurrently improving thermal hyperalgesia after PSNL [13]. In models involving neurogenic inflammation, Y1 agonists inhibit capsaicin evoked mechanical allodynia through a reduction in CGRP release from primary afferent terminals [58]. Thus the effects of NPY in the skin are extremely complex, and depend on the receptor on which it acts, the location of these receptors, and the model used.

Not all of the NPY immunoreactivity has been accounted for as many NPY-IR cell bodies and axons were not shown to co-express any of the markers used in this study. Future studies will be required to identify these primary afferents, however it is expected that they will likely possess medium to large diameter cell bodies and send central projections to lamina III and IV.

In conclusion, these results show novel and increased sources of NPY in the periphery in a rat model of trigeminal neuropathic pain. How exactly this increase in NPY contributes to pain should be the subject of future investigations.

## Methods

### Animals

Adult male Sprague-Dawley rats (250–350 g; Charles River, Canada) were maintained on a 12-h light/dark cycle and allowed access to food and water *ad libitum*. All protocols were approved by the McGill University Animal Care Committee and complied with the policies and guidelines outlined by the Canadian Council on Animal Care and the International Association for the Study of Pain.

### Surgical procedures

A modified chronic constriction injury [59], as described previously [3], was applied to the mental nerve of the rat, a purely sensory branch of the trigeminal nerve that innervates the rat lower lip [6]. Briefly, the animals were anesthetized with isofluorane and the mental nerves were bilaterally exposed from their point of exit from the mental foramina. Proximal to its branching, the nerve was freed from adhering tissue and 2 loose silk ligatures (8.0, Covidien), separated by approximately 2 mm, were tied around the nerve. The incision was closed with absorbable sutures (Vicryl, Ethicon). A sham surgery, where the mental nerve was visualized but no sutures were applied, was used as a control.

### Fluorogold injections

Because the trigeminal ganglion contains the sensory neurons from all three branches of the trigeminal nerve, Fluorogold was used to determine if the upregulation of NPY was occurring specifically in the cell bodies of mental nerve sensory neurons, or if it was occurring globally throughout the ganglion. Using a calibrated glass micropipette, 1 μl of 5 % Fluorogold solution (Fluorochrome, LLC) diluted in distilled water was injected directly into the mental nerve of sham and CCI animals, proximal to the first suture. As above, the incision was closed with absorbable sutures. Animals were sacrificed one week after injection.

### Behaviour: mechanical allodynia

All animals (n = 8/group) were tested 2–6 weeks following CCI surgery by a blinded experimenter. We used von Frey filaments applied to the lower lip to determine the 50 % withdrawal threshold to punctate mechanical stimulation. Each rat was placed in a transparent Plexiglas cage atop a wire mesh grid and was allowed to habituate to their surroundings for 30 min before behaviour testing. Von Frey filaments of increasing stiffness were applied perpendicularly, in the midline, to the lower lip skin following the up-and-down method described by Dixon [60] until a positive reaction, considered a brisk head withdrawal, was observed [7]. Following the first positive reaction, the next lighter filament was applied. If no reaction, the next stiffer filament was applied; if a reaction was observed, the next lighter filament was applied. After the first positive filament, four additional filaments were applied and the 50 % withdrawal threshold was calculated using the methods outlined by Chaplan *et al.* [61]*.* Mechanical allodynia was considered as a significant reduction in withdrawal threshold when compared to shams, as measured by a 1way analysis of variance (ANOVA) with Dunnett’s post hoc test. Statistical significance was set at p < 0.05.

### Tissue preparation

NPY expression in the trigeminal ganglion, the mental nerve and in the skin of the lower lip following CCI was visualized using immunohistochemistry. Rats were deeply anesthetized with Equithesin (0.3 mL/100 g) and perfused transcardially with 100 mL of perfusion buffer and 500 mL of 3 % paraformaldehyde and 15 % saturated picric acid (v/v) in 0.1 M phosphate buffer (PB), pH 7.4, for 30 min. Trigeminal ganglia, mental nerves and hairy lower lip skin were dissected out and postfixed for 1 h in the above fixative and cryoprotected in 30 % sucrose in PB for 24 h at 4 °C. Tissue was embedded in an optimum cutting temperature medium (Tissue Tek, OCT) and cut on a cryostat at − 20 °C. Parasagittal sections of mental nerve and horizontal sections of trigeminal ganglia were cut at a thickness of 14 μm and 20 μm, respectively, and mounted directly on slides. Sections of skin were cut at 50 μm and collected as free-floating in 0.01 M phosphate-buffered saline (PBS).

### Immunohistochemisty and lectin binding

To determine in which population of fibers NPY was expressed in after CCI, the mental nerves, trigeminal ganglia and skin were processed for immunohistochemistry using antibodies against NPY in combination with those against either neurofilament 200 (NF200), calcitonin gene-related peptide (CGRP) or dopamine β-hydroxylase (DBH) to label myelinated fibers, peptidergic sensory fibers and sympathetic fibers, respectively. The lectin IB4 was used to identify non-peptidergic sensory fibers. Fluorogold is a fluorescent dye and requires no additional staining. All sections were washed for 30 min with PBS containing 0.2 % Triton-X (PBS-T), incubated in 50 % ethanol for 30 min and washed in PBS-T. Depending on the species in which the secondary antibody was raised in, the tissue was either blocked in 10 % normal donkey or normal goat serum for 1 h. Primary antibodies were used at the following concentrations – anti-NPY (1:4000, Rabbit polyclonal, Abcam, ab10980, lot#GR16697-2), anti-NF200 (in skin and nerve, 1:5, mouse monoclonal, Abcam, ab910, lot# GR130394-2; in ganglia, 1:1000, mouse monoclonal, Sigma, N0142, lot# 053 M4756), anti-CGRP (1:1000, mouse monoclonal, Sigma, C7113, lot#032 M4862), anti-DBH (1:50, mouse monoclonal, Medimabs, MM-0008-P, lot#83120927-P) and IB4 conjugated to Alexa Fluor 568 (1:200, Molecular Probes, I21412, lot#880292) made in 5 % blocking serum (goat or donkey) in PBS-T and left to incubate overnight on the shaker at 4 °C. Following 30 min of washes with PBS-T, the tissue was incubated with the appropriate secondary antibody diluted in PBS-T for 2 h at room temperature– goat anti-rabbit conjugated to Alexa Fluor 488 (1:800, Molecular Probes, A11034, lot#1212189), goat anti-mouse conjugated to Alexa Fluor 568 (1:800, Molecular Probes, A11031, lot# 822389), or donkey anti-mouse conjugated to Rhodamine Red X (1:200, Jackson Immunoresearch, 715-296-151, lot#72430). Following 30 min of washes, free-floating lip sections were mounted on gelatin-subbed slides and all slides were coverslipped with Aqua Polymount (Polysciences).

Representative images were taken using a Zeiss LSM510 confocal microscope equipped with Ar and He-Ne lasers using either 10× dry, 40× water-immersion, or 63× oil-immersion objectives. For co-localization studies both z stack and single optical slice images were taken to ensure bona fide detection of co-localization between markers. In the figures, all images of the mental nerve and trigeminal ganglia are single plane images, with z stack images of the skin.

### Quantification

Images used for quantification were taken on a Zeiss Axioplan 2e imaging fluorescence microscope (Carl Zeiss Microscopy LLC), with either a 20× or a 40× objective. Images were acquired with a high-resolution color digital camera with Zeiss Axiovision 4.8 software.

### NPY fiber density quantification in the skin

Changes in NPY-IR innervation in the lower lip skin were determined by analyzing the density of fibers within the upper dermis, defined as the area above the opening of the sebaceous glands [6]. Six randomly chosen fields per lip section from 3 sections were captured, totaling 18 images per animal. Six animals were used for each time point. Quantification was performed using an MCID Elite image analysis system (Imaging Research Inc., St. Catharines, ON, Canada) to determine the total fiber length (mm) per unit area of upper dermis (mm^2^), as described by us previously [7]. Briefly, we used a function of the MCID software that was developed to specifically and accurately detect fibers. After detection, fibers were skeletonized to 1 pixel in width, and the total fiber length per unit area was determined by the software and compared using an ANOVA with Dunnett’s post hoc test, with p < 0.05 considered significant.

### Trigeminal ganglia quantification

To determine the changes in NPY expression in the trigeminal ganglia, 3 entire sections per ganglion (and per animal) were captured using the 20× objective. From these images, the numbers of NPY-IR cell bodies with visible nuclei were counted. In addition the diameter of NPY-IR cells was measured using the Axiovision software. The extent of colocalization between NPY and either Fluorogold, CGRP, IB4 or NF200, was determined by counting both the total number of cell bodies expressing each individual marker as well as those labelled with both markers. A percentage was calculated from these values and when applicable was compared using a Student *t*-test with p < 0.05 considered significant.

### Quantification of NPY/DBH colocalization in the skin

Three random sections per animal were visualized using the 40× objective from the fluorescence microscope and all of the DBH-IR and NPY-IR fibers in the upper dermis were counted, taking note of whether the fiber was also NPY-IR or DBH-IR, respectively. Total section length was measured in ImageJ. The number of DBH-IR fibers per section length (mm) as well as the percentage of DBH fibers expressing NPY or NPY fibers expressing DBH was calculated and in all cases was compared using a 2 way ANOVA with Bonferroni post-hoc analysis with p < 0.05 considered significant.
